# Supplementary material for: DNA replication dynamics are associated with genome composition in Plasmodium species
Source: Nucleic Acids Res. 2025 Feb 25;53(4):gkaf111. doi: 10.1093/nar/gkaf111 (PMC11851108; doi:10.1093/nar/gkaf111)

SUPPLEMENTARY FIGURE AND TABLE LEGENDS

Supplementary Figure 1. Plasmid maps used for tagging *P. knowlesi orc1* gene and subsequent genotyping and confirmation of successful tagging.
(A) Cas9 plasmid with sgRNA targeting *P. knowlesi* A1-H.1 *orc1* (PKA1H_130007800); (B) Vector containing the donor DNA with ~800 bp homology arms flanking the 3xHA tag followed by a skip peptide (T2A) and neomycin resistance (NeoR); (C) Genotyping of transfectants before (-) G418 and after (+) G418 selection using wild-type (WT) and mutant (M) directed primers; WT primers will give 917 bp and 1876 bp fragments for wild-type and mutant parasites, respectively; M primers will give a 1009 bp fragment in mutant parasites and none for wild-type parasites; (D) Western blot of fractionated protein lysates showing a majority of HA-tagged protein in the nuclear soluble fraction (NS). GAPDH and histone H4 are shown as control, present solely in the cytosolic (C) and insoluble nuclear fraction (NI), respectively. We noted a similar but more pronounced double banding of the probed tagged protein compared to what we have observed *P. falciparum* (8). This could be a result of a small N-terminal truncation, thus producing a slightly smaller band.

Supplementary Figure 2. Chromosomal distribution of DNAscent replication forks and origins in relation to *SICAvar*/*var* genes. Chromosome plots showing replication forks and origins in relation to *SICAvar* genes
(A) and fork density plots over *SICAvar* genes during mid-schizogony (B) in *P. knowlesi*. Similar plot showing distribution of replication forks and origins (C) and fork density over *var* genes in *P. falciparum* (D). On the chromosome plots, black bars represent *SICAvar*/*var* gene locations, blue bars represent replication origins during early schizogony origins, and green bars represent origins at mid-schizogony. Light blue and dark blue line plots above the chromosomes represent replication fork density during early and mid-schizogony, respectively. Light green and dark green line plots below the chromosomes represent replication fork density during early and mid-schizogony, respectively. Fork density plots show mean fork density over *SICAvar*/*var* genes ± 1 kb, and significant difference was calculated to compare fork density at the start versus at the end of the genes.

Supplementary Figure 3. *P. knowlesi* and *P. falciparum* ORC1-HA ChiP.
(A) The left fingerprint plot shows no enrichment in *P. knowlesi* ORC1-HA ChIP signal in comparison with the input. In contrast, *P. falciparum* ORC1-HA fingerprint plot shows clear difference in signal enrichment between ChIP and input. (B) Representative visualisation of ORC1-HA enrichment over chromosome 1 shows greater log2ratios in *P. falciparum* in comparison to *P. knowlesi*. MACS2* peak calling was able to call a total of 6785 ORC1-HA peaks and 8997 summits in *P. falciparum* at 30 hpi, while only 41 peaks and 61 summits were called in *P. knowlesi* at the equivalent timepoint, 22 hpi.

* Zhang, Y., Liu, T., Meyer, C.A., Eeckhoute, J., Johnson, D.S., Bernstein, B.E., Nusbaum, C., Myers, R.M., Brown, M., Li, W. *et al.* (2008) Model-based analysis of ChIP-Seq (MACS). *Genome Biol*, **9**, R137.

Supplementary Table 1. List of oligonucleotide and primer sequences used for tagging *P. knowlesi orc1* gene and subsequent genotyping. Sequences of primers used to amplify fragments to construct the donor DNA, guide RNA oligos, sequencing and genotyping primers.

Supplementary Table 2. Summary of the total number of replication forks and origins called by DNAscent in *P. falciparum* (8) and *P. knowlesi*.

Supplementary Table 3. Genome-wide correlation between the localisation of HP1 and DNAscent forks and origins.

Supplementary Table 4. Percentage identity matrices between human, *P. knowlesi* and *P. falciparum* (A) replisome proteins and (B) helicases (RECQ1 and WRN). Percentages are presented as a heatmap with colours going from blue to red representing percentage identity scores ranked from highest to lowest.

Supplementary Figure 1

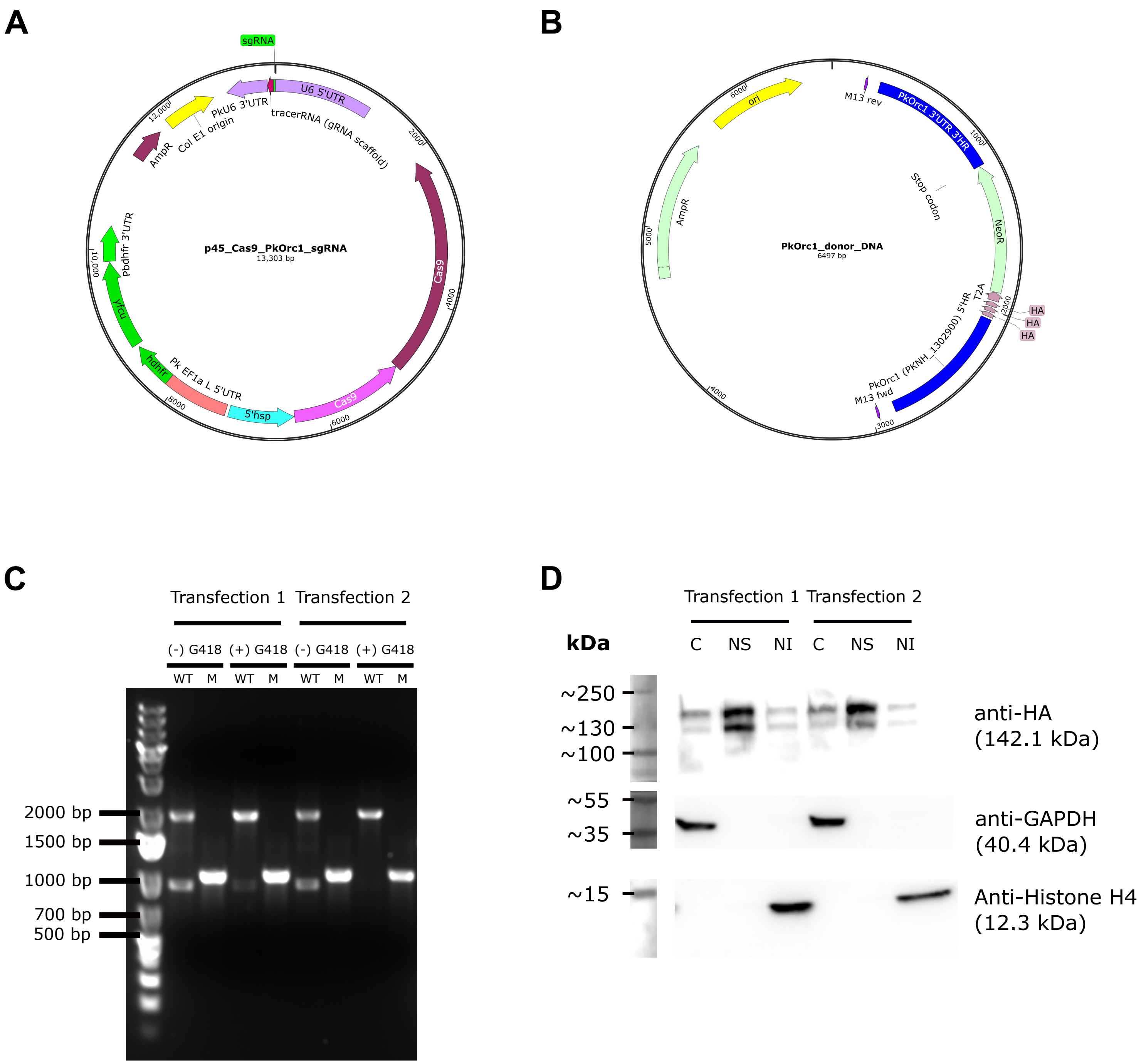

Supplementary Figure 2

A B

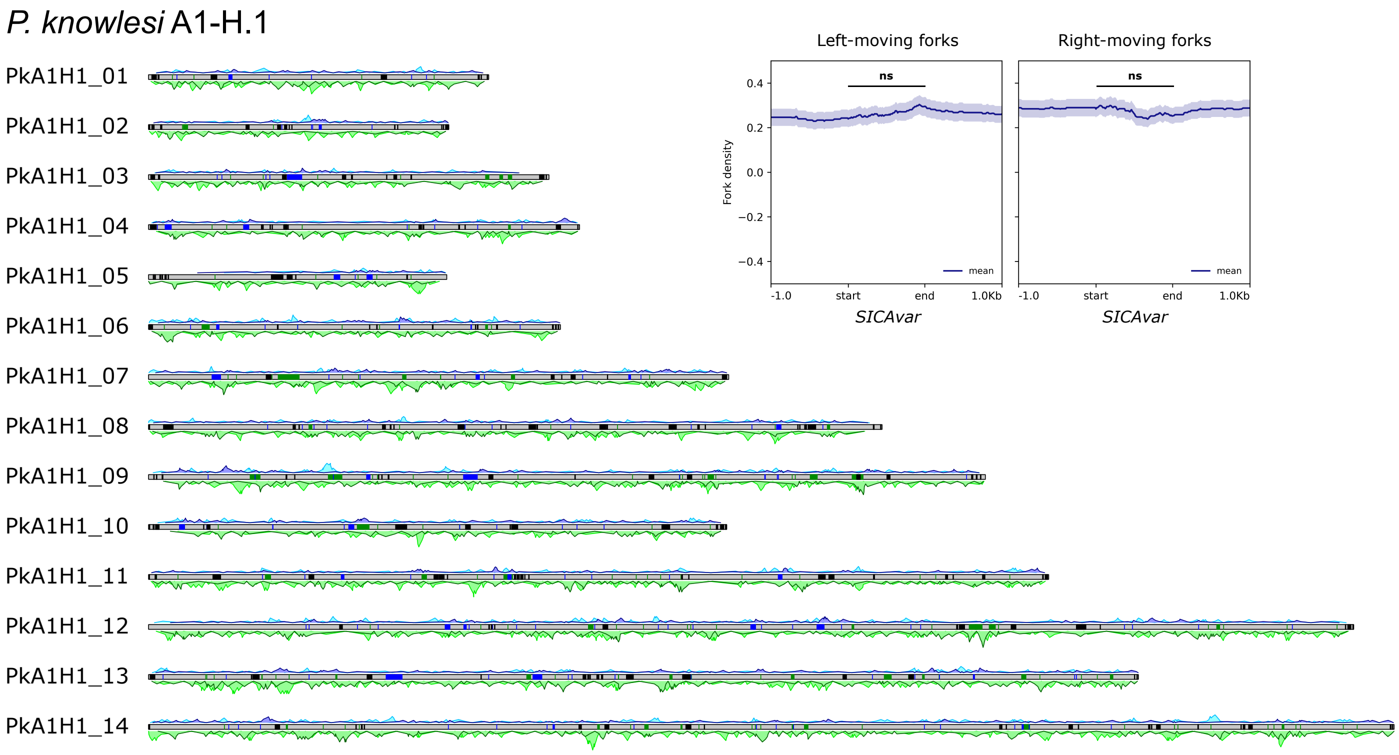

C D

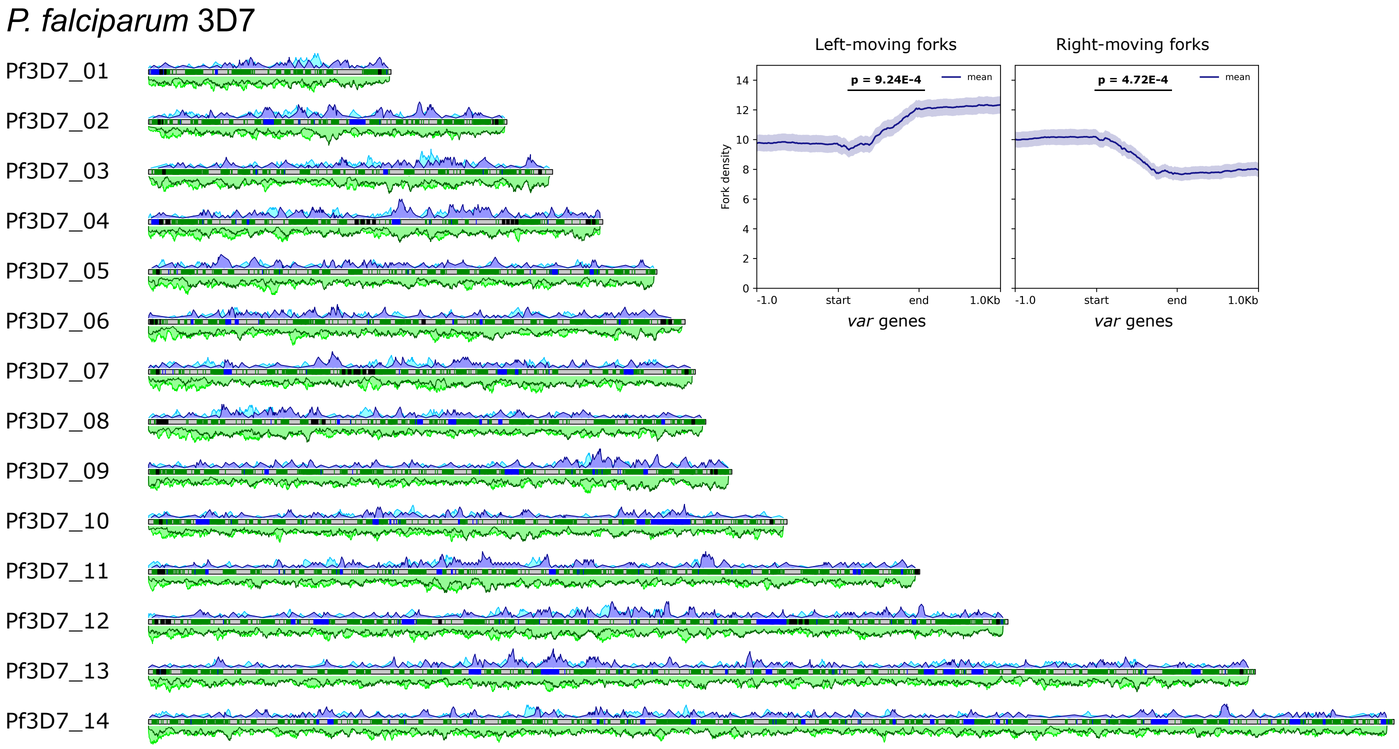

Supplementary Figure 3

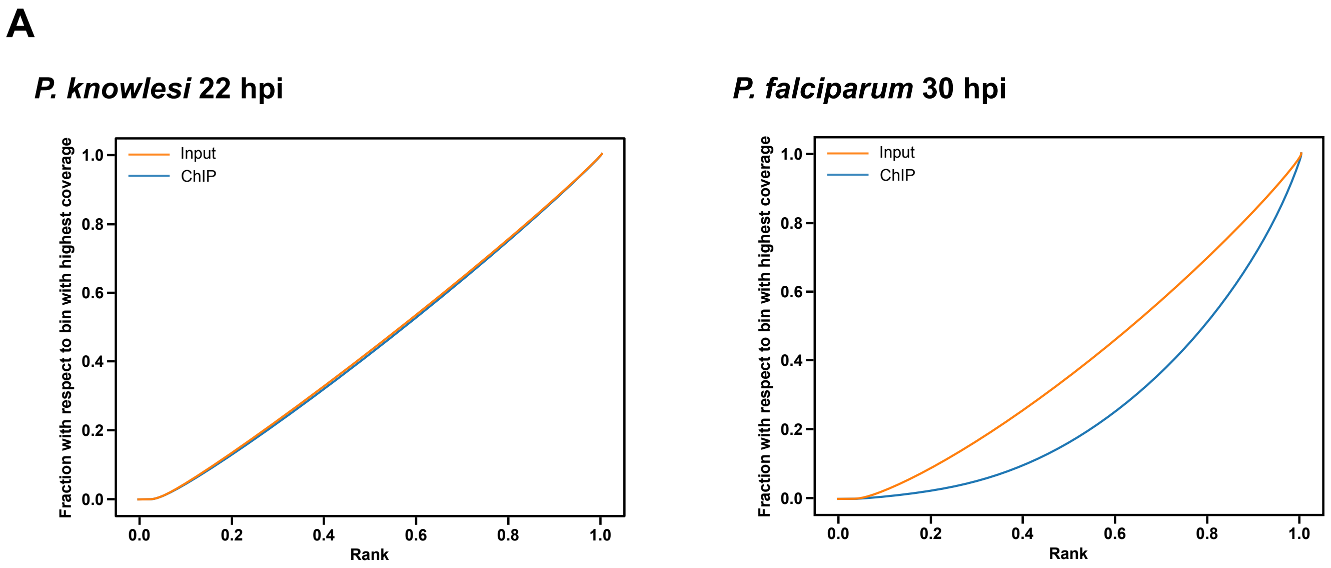

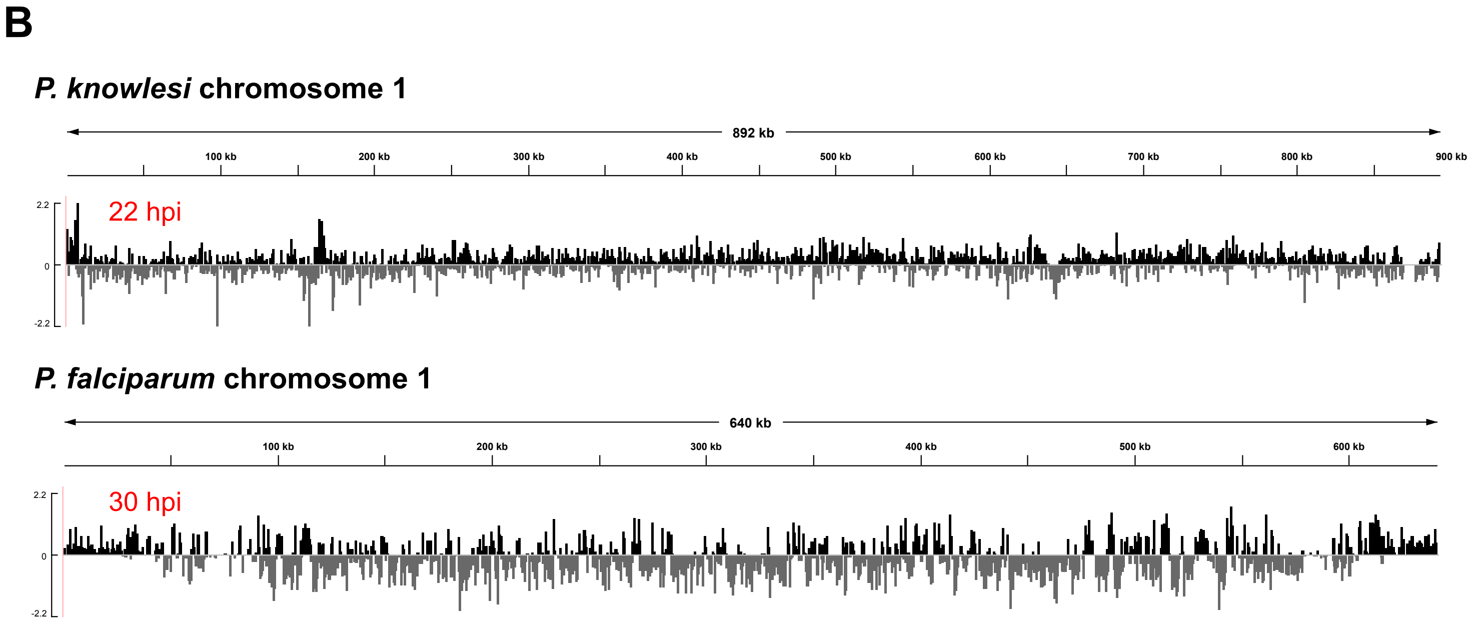

Supplement: gkaf111_Supplemental_Files [file gkaf111_supplemental_files.zip › Updated Supp.pdf]
